# Supplementary material for: Mass campaigns with antimalarial drugs: a modelling comparison of artemether-lumefantrine and DHA-piperaquine with and without primaquine as tools for malaria control and elimination
Source: BMC Infect Dis. 2015 Mar 22;15:144. doi: 10.1186/s12879-015-0887-y (PMC4376519; doi:10.1186/s12879-015-0887-y)
Supplement: Additional file 1: Table S1. — Compartmental model PK parameters. Table S2. PK parameter sourcing. Table S3. Scaling EIR by reducing larval habitat. Table S4. Correlations of prevalence after MDA campaigns with and without PQ. [file 12879_2015_887_MOESM1_ESM.pdf]

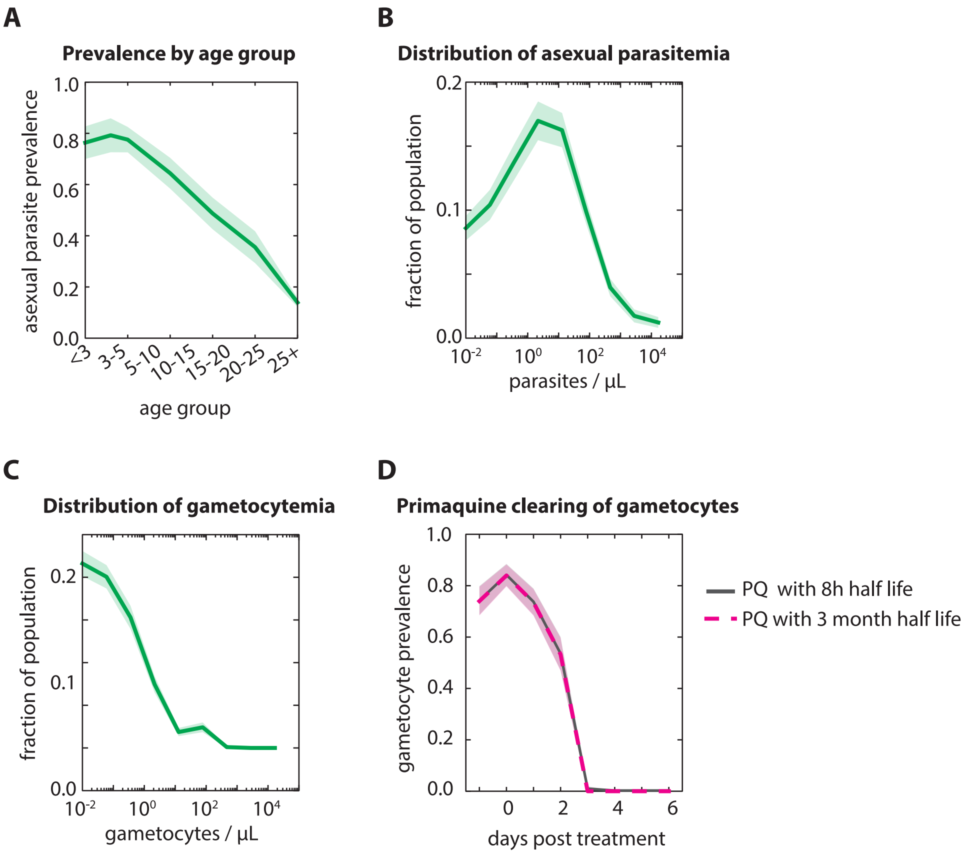

Figure S1. Asexual parasite prevalence and distribution of asexual parasitemia and gametocytemia under constant annual EIR 36 with a semi-immune population in the absence of intervention.

A. Age-dependent prevalence shows decreasing prevalence as individuals age. Shaded area: 95% confidence interval.

B. Distribution of asexual parasitemia. Shaded area: 95% confidence interval.

C. Distribution of gametocytemia. Shaded area: 95% confidence interval.

D. Comparison of gametocyte clearance with AL+PQ. PQ as parameterized in Figure 2 (gray) and with half life extended to 3 months (pink). Shaded area: 95% confidence interval.

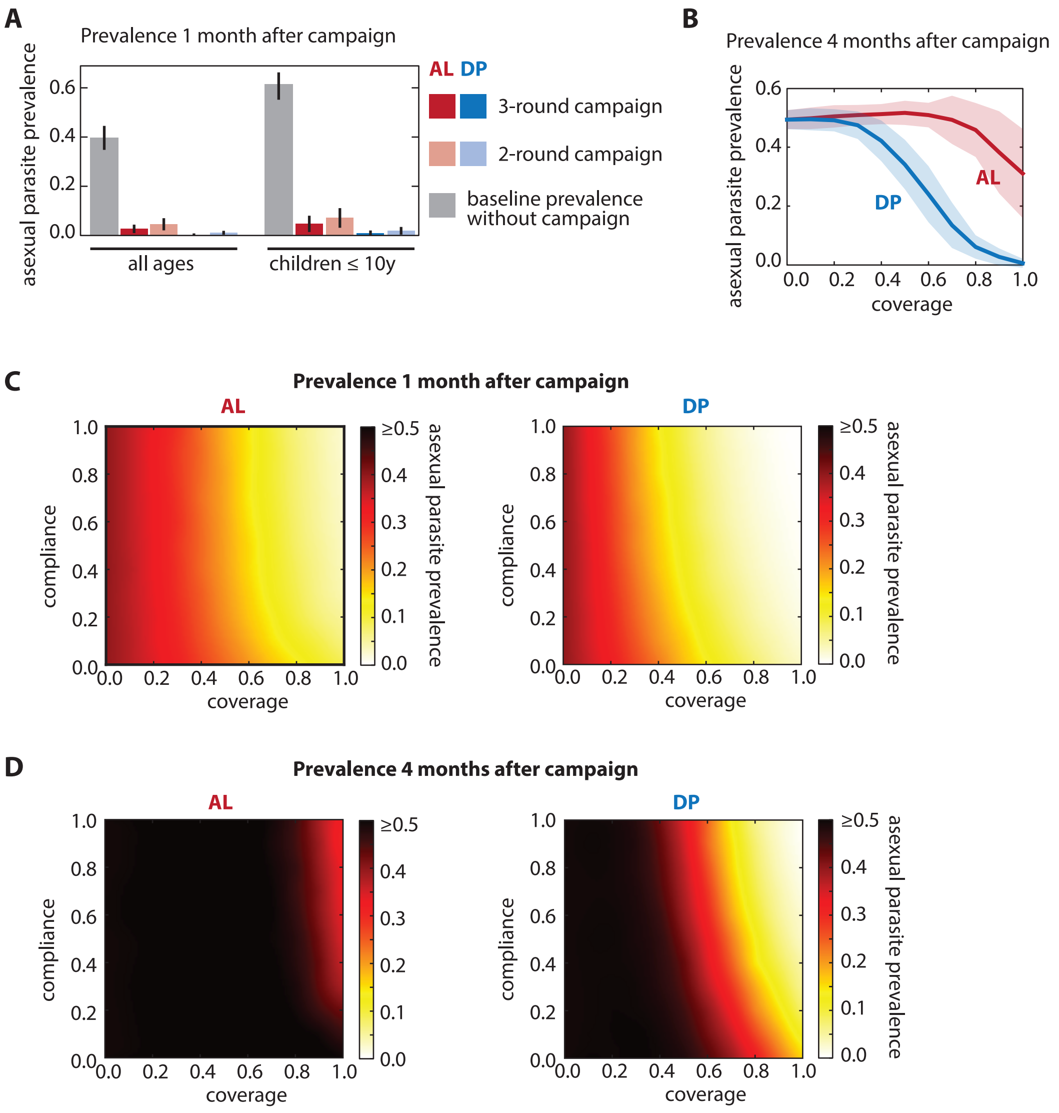

Figure S2. Campaign outcome dependence on timing, coverage, and compliance
A. Prevalence 1 month after campaign for 3-round and 2-round MDA campaigns with AL or DP, 100% coverage and 100% compliance. Annual EIR was 50. Error bars: 95% confidence interval.
B. Prevalence 4 months after 3-round MDA campaigns with varying coverage and 100% compliance. Annual EIR was 50. Shaded areas: 95% confidence interval.
C. Prevalence 1 month after campaign for 3-round MDAs with AL or DP, varying coverage and compliance. Annual EIR = 50.
D. Prevalence 4 months after campaign for 3-round MDAs with AL or DP, varying coverage and compliance. Annual EIR = 50.

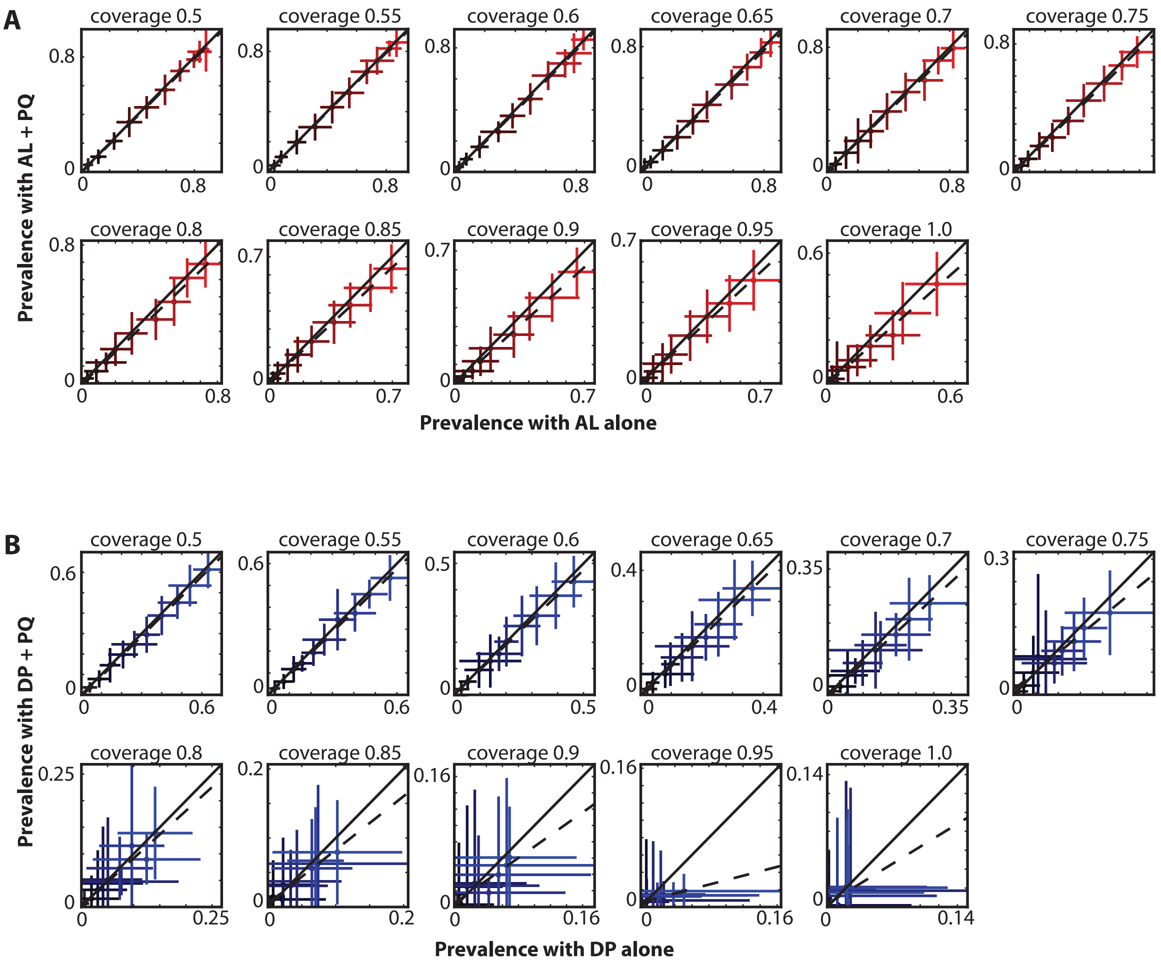

Figure S3. Prevalence 4 months after campaigns with and without primaquine.
Prevalence measured with PCR with sensitivity at 0.05 parasites/μL. EIR is sampled at constant coverage for 100 stochastic realizations for each EIR. Compliance is 100%. Mean +/- one standard deviation is shown for each EIR value. A linear regression is shown (dotted line) correlating prevalence after a 3-round MDA with AL/DP + PQ to prevalence after a 3-round MDA with AL/DP alone.
