# Supplementary material for: Mass campaigns with antimalarial drugs: a modelling comparison of artemether-lumefantrine and DHA-piperaquine with and without primaquine as tools for malaria control and elimination
Source: BMC Infect Dis. 2015 Mar 22;15:144. doi: 10.1186/s12879-015-0887-y (PMC4376519; doi:10.1186/s12879-015-0887-y)
Supplement: Additional file 2: Figure S1. — Asexual parasite prevalence and distribution of asexual parasitaemia and gametocytaemia under constant annual EIR 36 with a semi-immune population in the absence of intervention. Figure S2. Campaign outcome dependence on timing, coverage, and compliance. Figure S3. Prevalence 4 months after campaigns with and without primaquine. [file 12879_2015_887_MOESM2_ESM.docx]

**Supplementary Tables**

**Table S1: Compartmental model PK parameters.** Values are best-guess consensus values from literature review in Table S2. BW: body weight (kg)

|  |  | AM | LF | DHA | PPQ | PQ |
| --- | --- | --- | --- | --- | --- | --- |
| k_a_ | absorbance rate constant from gut to central compartment (1/h) | 0.5 | 0.2 | 0.37 | 0.5 | 2 |
| V_c_ | volume of distribution of central compartment (L) | 3.5*BW | 60*(BW^0.35^) | 3*BW | 20*BW | 4*BW |
| k_e_ | elimination rate constant from central compartment (1/h) | 2 | CL/V_c_ | 1 | CL/V_c_ | 0.12 |
| CL | clearance rate (from central compartment) (L/h) | 600 | 6 |  | 1.5*BW |  |
| V_p_ | volume of distribution of secondary compartment (L) | V_c_ | V_c_ |  | 22000 |  |
| k_12_ | distribution rate constant from central to secondary compartment (1/h) | CL/V_c_ | 0.001 |  | 0.19 |  |
| k_21_ | intercompartmental clearance from secondary to central compartment (1/h) | 0 | CL/V_p_ |  | 0.005 |  |
| k_ep_ | elimination rate constant from secondary compartment (1/h) | 1 | 4.8/V_p_ |  | 0 |  |

**Table S2: PK parameter sourcing**

| **Artemether** | | |
| --- | --- | --- |
| k_a_ (1/h) | 0.37 | Ezzet Br J Clin Pharm 1998 |
|  | 0.878 | Tarning Malaria J 2012 |
|  | 0.27 | Hodel Malaria J 2013 |
| V_c_ (L) | 217 | Ezzet Br J Clin Pharm 1998 |
|  | 2160 | Tarning Malaria J 2012 |
|  | 129 / kg | Hodel Malaria J 2013 |
| Distribution (1/h) | 5.86 | Hodel Malaria J 2013 |
| Elimination t_1/2_ (h) | 4.2 | Na Bangchang Br J Clin Pharm 1994 |
|  | 3.9 | Mwesigwa AAC 2010 |
|  | 0.84 | Ezzet Br J Clin Pharm 1998 |
|  | 1.77 | Tarning Malaria J 2012 |
| Clearance (L/h) | 180 | Ezzet Br J Clin Pharm 1998 |
|  | 875 | Tarning Malaria J 2012 |
|  | 24.7*BW^0.75^ | Hodel Malaria J 2013 |
| **Lumefantrine** | | |
| k_a_ (1/h) | 0.17 | Ezzet AAC 2000 |
|  | 0.13 | Ezzet Br J Clin Pharm 1998 |
|  | 0.54 | Hodel Malaria J 2013 |
| V_c_ (L) | 103 | Ezzet AAC 2000 |
|  | 213 | Ezzet Br J Clin Pharm 1998 |
|  | 59.9*BW^0.35^ | Hodel Malaria J 2013 |
| Distribution (1/h) | 0.114 | Ezzet AAC 2000 |
|  | 0.0065 | Ezzet Br J Clin Pharm 1998 |
|  | 0.00037 | Hodel Malaria J 2013 |
| Elimination t_1/2_ (h) | 76.8 | Ezzet AAC 2000 |
|  | 98.4 | Djimde Malaria J 2009 |
|  | 108 | Ezzet Br J Clin Pharm 1998 |
| Clearance (L/h) | 15 | Ezzet Br J Clin Pharm 1998 |
|  | 0.84*BW^0.52^ | Hodel Malaria J 2013 |
| k_21_ (1/h) | 0.015 | Ezzet AAC 2000 |
|  | 0.013 | Ezzet Br J Clin Pharm 1998 |
| **Dihydroartemisinin** | | |
| k_a_ (1/h) | 0.37 | Ezzet Br J Clin Pharm 1998 |
| V_c_ (L) | 106 | Ezzet Br J Clin Pharm 1998 |
|  | 3.03*BW | Tarning AAC 2012 |
|  | 1.47*BW | Nguyen AAC 2009 |
| Elimination t_1/2_ (h) | 1.16 | Tarning AAC 2012 |
|  | 0.85 | Nguyen AAC 2009 |
| Clearance (L/h) | 169 | Ezzet Br J Clin Pharm 1998 |
|  | 1.72*BW | Tarning AAC 2012 |
|  | 1.19*BW | Nguyen AAC 2009 |
| **Piperaquine** | | |
| k_a_ (1/h) | 0.083 | Hung Br J Clin Pharm 2003 |
|  | 0.717 | Tarning AAC 2008 |
|  | 0.93 | Hodel Malaria J 2013 |
| V_c_ (L) | 14.5*BW | Hung Br J Clin Pharm 2003 |
|  | 8660*(1+0.0273*(BW-48)) | Tarning AAC 2008 |
|  | 829*BW [total V] | Tarning AAC 2012 |
|  | 346*BW | Hodel Malaria J 2013 |
| V_p_ (L) | 24000 | Tarning AAC 2008 |
|  | 18600 | Hodel Malaria J 2013 |
| Distribution (1/h) | 0.19 | Hung Br J Clin Pharm 2003 |
| Elimination t_1/2_ (h) | 543 | Hung Br J Clin Pharm 2003 |
|  | 667.2 | Tarning AAC 2008 |
|  | 576 | Tarning AAC 2012 |
|  | 427 | Nguyen AAC 2009 |
| Clearance (L/h) | 0.90*BW | Hung Br J Clin Pharm 2003 |
|  | 66*(1+0.0262*(BW-48)) | Tarning AAC 2008 |
|  | 1.32*BW | Tarning AAC 2012 |
|  | 4.5*BW^0.75^ | Hodel Malaria J 2013 |
| k_21_ (1/h) | 0.0056 | Hung Br J Clin Pharm 2003 |
| Q (L/h) | 131 | Tarning AAC 2008 |
|  | 122 | Hodel Malaria J 2013 |
| **Primaquine** | | |
| k_a_ (1/h) | 2.18 (children) | Moore AAC 2013 |
| V_c_ (L) | 5.1*BW | Na Bangchang Trans R Soc Trop Med Hyg 1994 |
|  | 4.8*BW | Na Bangchang Trans R Soc Trop Med Hyg 1994 |
|  | 4.59*BW | Binh Am J Trop Med Hyg 2009 |
|  | 3.42*BW | Binh Am J Trop Med Hyg 2009 |
|  | 46.7 | Kim Arch Pharm Res 2004 |
|  | 2921 | Bhatia Eur J Clin Pharm 1986 |
| Elimination t_1/2_ (h) | 6.3 | Na Bangchang Trans R Soc Trop Med Hyg 1994 |
|  | 6.2 | Na Bangchang Trans R Soc Trop Med Hyg 1994 |
|  | 6.1 | Binh Am J Trop Med Hyg 2009 |
|  | 6.8 | Binh Am J Trop Med Hyg 2009 |
|  | 3.76 | Kim Arch Pharm Res 2004 |
|  | 5.6 | Bhatia Eur J Clin Pharm 1986 |
|  | 4.9 | Edwards Br J Clin Pharm 1993 |
| Clearance (L/h) | 0.53*BW | Na Bangchang Trans R Soc Trop Med Hyg 1994 |
|  | 0.51*BW | Na Bangchang Trans R Soc Trop Med Hyg 1994 |
|  | 0.55*BW | Binh Am J Trop Med Hyg 2009 |
|  | 0.31*BW | Binh Am J Trop Med Hyg 2009 |
|  | 9.85 | Kim Arch Pharm Res 2004 |
|  | 37.6 | Bhatia Eur J Clin Pharm 1986 |
|  | 19.4 | Edwards Br J Clin Pharm 1993 |

**Table S3: Scaling EIR by reducing larval habitat**

| Larval habitat scale factor | EIR |
| --- | --- |
| 1.0 | 52 |
| 0.9 | 47 |
| 0.8 | 44 |
| 0.7 | 35 |
| 0.6 | 29 |
| 0.5 | 25 |
| 0.4 | 18 |
| 0.3 | 13 |
| 0.2 | 8 |
| 0.1 | 3 |
| 0.05 | 1 |

**Table S4: Correlations of prevalence after MDA campaigns with and without PQ.** Means and standard deviations of slopes and r^2^ values for linear regressions of prevalences measured 4 months after campaign with sensitivity 0.05 parasites/µL, 100 stochastic realizations, bootstrapped at 1000 resamples.

|  | AL + PQ vs AL alone | | | | DP + PQ vs DP alone | | | |
| --- | --- | --- | --- | --- | --- | --- | --- | --- |
| Coverage | Slope | Slope σ | r^2^ | r^2^ σ | Slope | Slope σ | r^2^ | r^2^ σ |
| 0.5 | 0.997 | 0.0085 | 0.999 | 0.0008 | 0.975 | 0.0124 | 0.999 | 0.0010 |
| 0.55 | 0.993 | 0.0085 | 0.999 | 0.0007 | 0.967 | 0.0150 | 0.998 | 0.0022 |
| 0.6 | 0.990 | 0.0096 | 0.999 | 0.0009 | 0.925 | 0.0195 | 0.998 | 0.0023 |
| 0.65 | 0.977 | 0.0091 | 0.999 | 0.0005 | 0.955 | 0.0263 | 0.997 | 0.0036 |
| 0.7 | 0.975 | 0.0109 | 0.999 | 0.0011 | 0.912 | 0.0329 | 0.997 | 0.0037 |
| 0.75 | 0.981 | 0.0102 | 0.999 | 0.0009 | 0.798 | 0.0441 | 0.986 | 0.0184 |
| 0.8 | 0.955 | 0.0134 | 0.998 | 0.0023 | 0.851 | 0.0549 | 0.968 | 0.0372 |
| 0.85 | 0.938 | 0.0136 | 0.999 | 0.0011 | 0.773 | 0.0702 | 0.973 | 0.0333 |
| 0.9 | 0.924 | 0.0157 | 0.998 | 0.0023 | 0.607 | 0.0905 | 0.914 | 0.0998 |
| 0.95 | 0.935 | 0.0204 | 0.998 | 0.0021 | 0.448 | 0.1341 | 0.869 | 0.1542 |
| 1.0 | 0.871 | 0.0222 | 0.997 | 0.0037 | 0.647 | 0.3033 | 0.790 | 0.2516 |
